# Research of the crankshaft high cycle bending fatigue experiment design method based on the modified unscented Kalman filtering algorithm and the SAFL approach

**DOI:** 10.1371/journal.pone.0291135

**Published:** 2023-09-12

**Authors:** Shuyang Rui, Dongdong Jiang, Songsong Sun, Xiaolin Gong

**Affiliations:** 1 College of Automobile and Traffic Engineering, Nanjing Forestry University, Nanjing, 210037, China; 2 Li Auto Vehicle Control Operation System, Hangzhou, 310000, China; Shandong University of Technology, CHINA

## Abstract

In modern engineering application, enough high cycle bending fatigue strength is the necessary factor to provide the basic safety security for the application of the crankshaft in automobile engines (both diesel and gasoline types). At present, this parameter is usually obtained through the standard bending fatigue experiment process, which is time consuming and expensive. In this paper, a new accelerated crankshaft bending fatigue experiment was proposed step by step. First the loading procedure was quickened through the prediction of the residual fatigue life based on the UKF (unscented Kalman filtering algorithm). Then the accuracy of the predictions was improved based on the modified sampling range and the theory of fracture mechanics. Finally the statistical analysis method of the fatigue limit load was performed based on the above predictions. The main conclusion of this paper is that the proposed accelerated bending fatigue experiment can save more than 30% of the bending fatigue experiment period and provide nearly the same fatigue limit load analysis result. In addition, compared with the particle filtering algorithm method, the modified UKF can provide much higher accuracy in predicting the residual bending fatigue life of the crankshaft, which makes this method more superior to be applied in actual engineering.

## 1. Introduction

At present, high output engines have been widely applied in all walks of life. Among the main parts of this equipment, the crankshaft is usually designed to provide high enough strength property due to its own function and working condition [1,2]. On the other hand, the new techniques such as the turbocharging approach applied on it makes the strength requirements more critical [3,4]. Thus, correct design and accurate evaluation of the strength property becomes indispensable for this part.

For this type of problems, continual research has been carried out in recent years. Up to now, there are generally two kinds of research aims in all [5,6]. The first type focus on researching the fatigue damage mechanism of the components, based on this the corresponding theoretical direction can be pointed out for the consequent improvement of the part. According to this research aim, Wang analyzed a broken crankshaft from pump by exampling the fracture surface and found out that the absence of a surface hardening treatment would result in the reduction of the fatigue strength, and the thread root between the smooth crankpin and the fillet will result in much higher stress concentration amplitude [7]. Aliakbari also chose a broken crankshaft from a heavy truck to be the object of study and discovered that the stress field around the lubricating hole was much less than that around the crankpin fillet, but other factors including the downshifting, the low hardness of the surface and the cluster impurities will also result in the unusually fracture at this location [8]. Fonte researched the failure mechanism of a crankshaft from a selected diesel engine applied on a motorcycle, and pointed out that although the part itself was properly designed, but the main journals were misarranged and generated the weakness of design close to the gear, as well as the crack initiation at the same place [9]. Infante conducted several kinds of standard tests on the broken crankshaft applied on a helicopter engine, the main conclusions carried out from these tests was that the failure reason of this component was not from the part itself, but the from damaged shell bearings assembled at the main journal [10]. Karim also conducted different experiments and tests to analyze the failure mechanism of the chosen ductile iron crankshaft, the results showed that the low crankpin hardness and the low nodularity of the material are the two main reasons for the failure of the part [11]. Tian analyzed the fracture crankshaft from a sport utility vehicle and discovered that the density of inclusions was great enough to act as the initiation of cracks [12]. Hosseini applied the acoustic emission method in detecting the fatigue crack of the crankshaft and proposed the corresponding entropy model, which could obviously reduce the data volume [13]. While for the second type, the research aim is to build the relationship model between the load amplitude applied on the given component and the corresponding fatigue life or some other parameters such as the fatigue safety factor, among these Gomes adopted the Soderberg criterion in researching the fatigue property of a type of crankshaft applied on the maritime engines, in this way the strength of the part was enhanced obviously [14]. Macek defined a new fatigue loading parameter by examining the broken fracture surface of the selected crankshaft, in this way the relationship between the fatigue life and the ratio of the maximum stress is carried out [15]. Bulut analyzed the load cycle of the selected crankshaft applied on a single cylinder engine, as well as the stress evolution caused, in this way the comprehensive evaluation of the part safety can be conducted based on the proposed parameter [16]. Singh researched the least fatigue life of the selected crankshaft based on the 3D finite element simulation result, as well as the influence of the fillet radius, based on these the optimal design of the part structures can be proposed [17]. Jose Wilmar analyzed the fatigue failure phenomenon of the loom crankshafts in textile industry and proposed both the static and fatigue safety factors, based on which the failure reasons of this part can be proposed in detail [18]. Liu also researched the fatigue property of a selected kind of crankshaft and proposed corresponding structure optimization to improve the strength [19].

Up to now, most of the fatigue property research of the component should be verified through corresponding experiment [20]. For the crankshaft, the most commonly used experiment equipment is the resonant bending fatigue experiment test bed [21,22]. This type of equipment can simulate nearly the same loading condition of the part during its working period, but the cost of the experiment is usually expensive due to the high number of the load cycles. In addition, the fatigue test results usually shows obvious divergence, which means the test samples should be large enough for the statistical analysis. As a result of this, the long experiment period will lead to huge economic cost.

In previous study, the particle filtering algorithm was applied in this topic, in this way the loading time of the experiment can be shortened to save time and money [23,24], but the predictions usually contains obvious errors (sometimes the relative error is more than 20%). In this paper, the UKF (unscented Kalman filtering algorithm) was chosen to predict the fatigue failure time node during the experiment, in this way the loading procedure was quickened obviously. Then the precision of prediction was improved with the help of the combination of the new sampling ranges and the theory of fracture mechanics. Finally the SAFL (statistical analysis of the fatigue limit) was adopted in analyzing the relationship between the predicted fatigue life and the load amplitude. The main conclusion of the above research is that based on the proposed method in this paper, about 30% of the loading period during the experiment process can be saved, and the fatigue limit load analysis result based on predictions is nearly the same with that based on the original experiment results (the relative error is less than 1.5%), which makes the application of this approach quite beneficial in actual engineering.

## 2. Method

### 2.1 The experiment process

At present, the crankshaft bending fatigue tests are carried out according to the industrial standard (the serial number is QC-T637-2000). As shown in Table 1, the whole experiment process is mainly composed by three parts: the load calibration stage, the loading stage, and the statistical analysis stages. Among these three stages, most of the experiment time is spent on the loading stage. In addition, the load rate within the loading stage mainly holds constant, which means that the time of the loading stage is directly determined by the load cycles [25]. As a result of this, the key technique in quicken the experiment process is to make sure when the crankshaft is able to be considered broken during the loading stage as early as possible.

**Table 1:** The time spans of each experiment stage.

Stage	Time span
Load calibration	Less than 0.5 day
Loading	More than 30 days
Statistical analysis	Less than 0.5 hour

**Fig 1:**
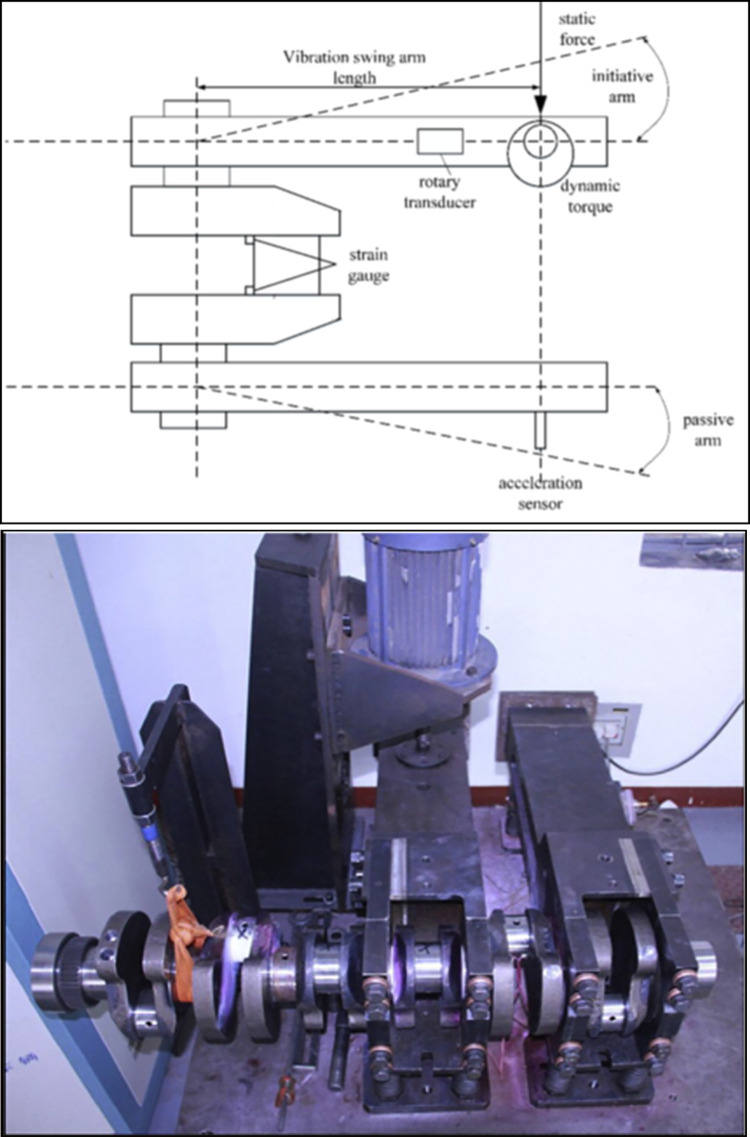
The experiment system (a. The schematic diagram, b. The physical photo).

Fig 1 shows the main components of the crankshaft bending fatigue test bed. The selected crankpin was fixed by the forked arms. During the loading stage, the rotating eccentric with the motor generates dynamic torque on the initiative arm, in this way the fixed crankpin will continuously suffer the cyclic bending moment. The amplitude of the load can be controlled accurately by monitoring the rotating speed of the motor. According to previous study, obvious stress concentration will happens at the crankpin fillet location [18]. As a result of this, the fatigue crack will occur at the same place and result in the reduction of the first order inherent frequency of the system. When the reduction amount of this parameter has reached 1Hz, the crankpin is judged to be broken [26,27].

### 2.2 The residual fatigue life prediction approach

As introduced in the above chapter, the acceleration of the loading stage is achieved based on the prediction of the failure time node during this stage. At present, the residual life prediction and some related health monitoring research has been carried out based on various kinds of models and methods [28–32], but the object of prediction among these researches are usually the working life or some other parameters, not the fatigue life. In the published related studies, most of the fatigue life predictions are carried out based on the S-N curve of the material. This method can conveniently determine the fatigue life of the smooth specimen under the given load, but the results always contains obvious errors due to the dispersion property of the fatigue experiment results, especially for the high cycle fatigue components (the errors may be more than double) [33,34]. In addition, as mentioned in the published works, the crankshaft usually shows obvious multi-axial stress property even though the type of the load applied on it is uniaxial, which result in the extra difficulty in determining the S-N curve of them [35–37]. In previous study, the particle filtering algorithm was applied in predicting the fatigue failure time node crankshaft during the bending fatigue test. This method can realize the goal in some occasions, but sometimes the errors are relatively large (more than 20%), which may be attributed to the particle degeneracy property of the method itself.

According to the theory of fatigue and reliability, for the given component under the cyclic loading condition, the fatigue damage accumulative process shows obvious nonlinear characteristic. In recent years, some experts came up with several methods in researching these problems, among these the UKF (unscented Kalman filtering algorithm) is considered to be an effective choice. Up to now, the application objects of this method are usually the working life of the lithium batteries and bearings [38,39]. The failure process of these components during the working is similar to that of the fatigue damage accumulation process. From this, this approach was chosen in this paper to predict the residual fatigue life of the selected type of crankshaft. The detailed main components of the crankshaft material are shown in Table 2.

**Table 2:** The main components of the crankshaft material.

Composition	Percentage%
C	0.38–0.45
Si	0.17–0.37
Mn	0.5–0.8
S	≤0.035
P	≤0.035
Gr	0.9–1.2
Ni	≤0.3
Cu	≤0.3
Mo	0.15–0.25

As shown in Table 2, the material of this crankshaft is 42CrMo, a typical kind of high strength alloy steel. As introduced in the previous chapter, the failure criteria of the crankshaft high cycle bending fatigue is defined according to the variation of the first order inherent frequency. So the residual fatigue life of the crankshaft is just the numbers of load cycles between the given variation and the failure variation of the first order inherent frequency. In this paper, the type of the empirical model selected in analyzing the relationship between the load cycles and the variation of the first order inherent frequency is the double exponential model, which can be expressed as:

y=aexp(bx)+cexp(dx) (2-1)

As shown in Eq (2-1), *x* is the load cycles, *y* is the decrement of the first order inherent frequency, *a*,*b*,*c*,*d* are all model parameters which can be determined by fitting the experimental data set. In this model, there are four parameters in all, which may result in the difficulty in solving the function. So in this paper, the model was simplified as:

$$Z(k+1)=\frac{\left(c(k)*\exp(d(k)*(t‐1))*(1‐\exp(b(k)‐d(k)))\right)}{\left(1‐\exp(b(k))\right)}+\alpha (k)+v(k)$$ (2-2)

where *Z*(*k*+1) is the observed value of the variation of the first order inherent frequency at the (*k+*1*)th* alternating load node, *α*(*k*) is the empirical value of the same parameter at the *kth* alternating load node, *v*(*k*) is the process noise which is distributed according to Gaussian model (the mean value is zero and the variance is *σ*_*w*_). Based on this simplification method, the four parameter fitting problem is converted to be the nonlinear one-dimensional equation of transfer. In this way the calculated amount and the time can be saved due to the reduced complexity.

According to the definition of the UKF method, the nonlinear system can be defined as:

$$\{\begin{array}{c} \delta (k+1|k)=\delta (k)+W(k)\\ \delta (k)={\left[b(k)\;c(k)\;d(k)\right]}^{T} \end{array}$$ (2-3)

where *δ*(*k*+1|*k*) is the observed value at the kth alternating load node, *δ*(*k*) is the estimated value at the previous alternating load node. The sigma points at this time node can be determined based on the state estimated values and the corresponding systematic error covariance matrix, the results can be expressed as:

$$\left\{ \begin{array}{l} \delta^{{}_{{}^{\left(0\right)}}}=\bar{\delta },i=0\\ \delta^{{}_{{}^{\left(i\right)}}}=\bar{\delta }+{\left(\sqrt{(n+\lambda )P}\right)}_{i},i=1∼n\\ \delta^{{}_{{}^{\left(i\right)}}}=\bar{\delta }-{\left(\sqrt{(n+\lambda )P}\right)}_{i},i=n+1∼2n \end{array}\right.$$ (2-4)

where *λ* is the scale factor and *n* is the dimension of the state variable. The weight coefficient of each point is defined as:

$$\left\{ \begin{array}{l} \omega_{m}^{\left(0\right)}=\frac{\lambda }{n+\lambda }\\ \omega_{c}^{\left(0\right)}=\frac{\lambda }{n+\lambda }+(1-\alpha^{2}+\beta )\\ \omega_{m}^{\left(i\right)}=\omega_{c}^{\left(i\right)}=\frac{\lambda }{2(n+\lambda )},i=1∼2n \end{array}\right.$$ (2-5)

in Eq (2-5), *i* is the number of the sigma points, *ω*_*m*_ and *ω*_*c*_ are the weight coefficients of the mean value and the variance respectively. By combining these two equations above, the nonlinear mapping of these sigma points can be expressed as:

$$\{\begin{array}{c} \delta^{\left(i\right)}(k+1|k)=f[k,\delta^{\left(i\right)}(k|k)]=\sum_{i=0}^{2n}\omega^{\left(i\right)}\delta^{\left(i\right)}(k+1|k)\\ S(k+1|k)=\sum_{i=0}^{2n}\omega^{\left(i\right)}[\hat{\delta }(k+1|k)-\delta^{\left(i\right)}(k+1|k)][\hat{\delta }(k+1|k)-\delta^{\left(i\right)}(k+1|k){]}^{T} \end{array}$$ (2-6)

As shown in Eq (2-6), *δ*^(*i*)^(*k*+1|*k*) is the predicted value and *S*(*k*+1|*k*) is the covariance matrix. Based on these parameters, the new sigma point set can be proposed with the help of the unscented transform method, the result can be expressed as:

$$\delta^{\left(i\right)}(k+1|k)=[\begin{array}{ccc} \hat{\delta }(k+1|k) & \hat{\delta }(k+1|k)+\sqrt{\left(n+\lambda )S(k+1|k\right)} & \hat{\delta }(k+1|k)-\sqrt{\left(n+\lambda )S(k+1|k\right)} \end{array}]$$ (2-7)

By taking these new sigma points into the observation and prediction equations, the mean value and the covariance of the predictions can be determined as:

$$\left\{ \begin{array}{l} S_{z_{k}z_{k}}=\sum_{i=0}^{2n}\omega^{\left(i\right)}[Z^{\left(i\right)}(k+1|k)-\bar{\zeta }(k+1|k)][\zeta^{\left(i\right)}(k+1|k)-\bar{\zeta }(k+1|k){]}^{T}+R\\ S_{x_{k}z_{k}}=\sum_{i=0}^{2n}\omega^{\left(i\right)}[\delta^{\left(i\right)}(k+1|k)-\bar{\zeta }(k+1|k)][\delta^{\left(i\right)}(k+1|k)-\bar{\zeta }(k+1|k){]}^{T} \end{array}\right.$$ (2-8)

Finally the Kalman gain matrix and the system status and covariance updates can be determined as:

$$\{\begin{array}{l} K(k+1)=S_{x_{k}z_{k}}S_{z_{k}z_{k}}^{-1}\\ \hat{\delta }(k+1|k+1)=\hat{\delta }(k+1|k)+K(k+1)[\zeta (k+1)-\hat{\zeta }(k+1|k)]\\ S(k+1|k+1)=S(k+1|k)-K(k+1)S_{z_{k}z_{k}}K^{T}(k+1) \end{array}$$ (2-9)

**Fig 2:**
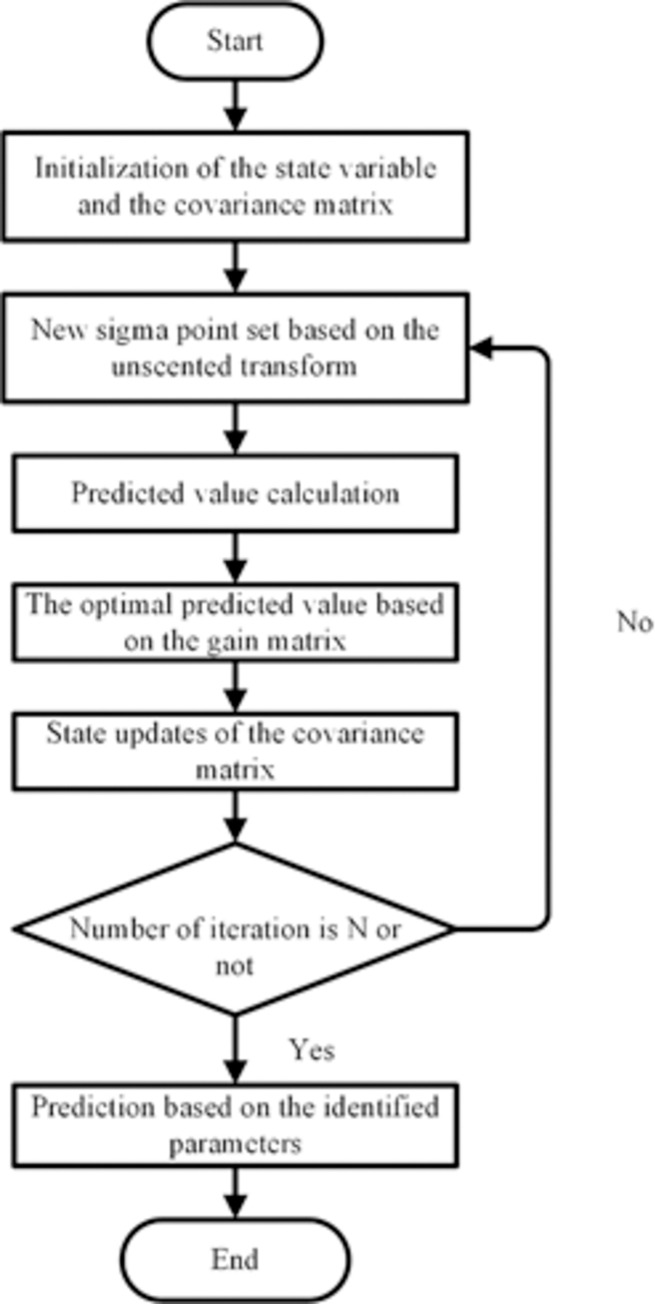
The research process of the UKF method.

The detailed process of the prediction is shown in Fig 2.

### 2.3 The statistical analysis method

**Fig 3:**
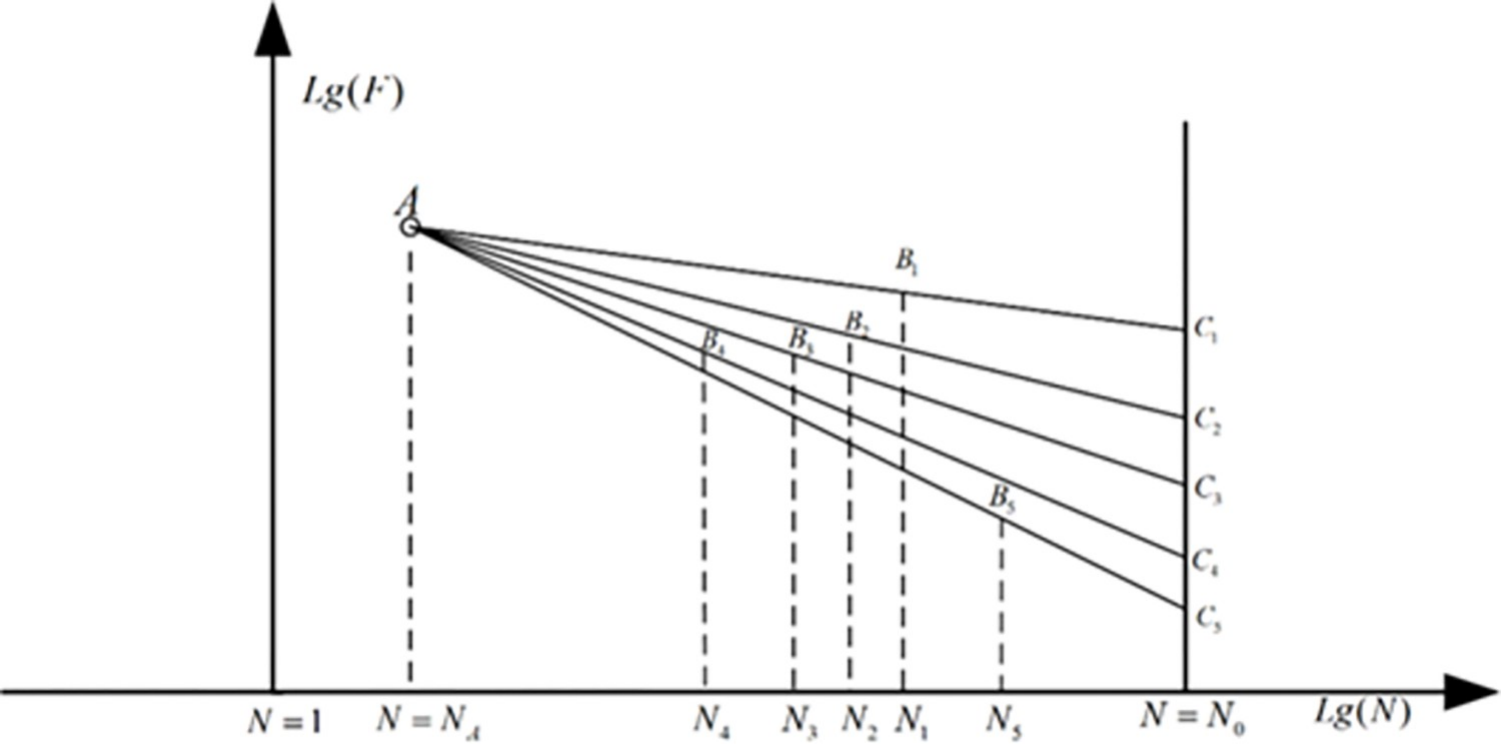
The load-life relationship in the couple log coordinate.

As mentioned in the former chapter, usually great dispersion exists in the test results of the fatigue experiment, especially for the high cycle fatigue test of the solid parts. On the other hand, the service life of a crankshaft is limited to a certain number of cycles depending on the demand of the travelling distance [40–42]. As a result of this, compared with the common fatigue property evaluation parameter (usually the fatigue life under a given load), it is more important to correctly evaluate the high-cycle fatigue load of a crankshaft under a specified fatigue life. So for the engineering parts such as the crankshafts, it’s necessary to apply professional statistical analysis method in analyzing the load-life relationship. At present, the most popular method applied in this field is the SAFL (statistical analysis for the fatigue limit) method. The fundamental theory of this method is shown in Fig 3 [43].

As shown in Fig 3, a point A exists in the couple log coordinate under the low fatigue life cycle (usually 1000). The load amplitude of this point is determined by the direct linear fitting of the experiment results. The fatigue limit load amplitude distributed in each case in determined by mapping the experiment result in each case from the point A to the specified fatigue life (for the crankshaft made by high strength steels, this parameter is 10^7^). The relationship between them can be expressed as:

$$\mathrm{lg}C_{i}=\mathrm{lg}S_{A}\frac{\mathrm{lg}N_{0}-\mathrm{lg}N_{i}}{\mathrm{lg}N_{A}-\mathrm{lg}N_{i}}+\mathrm{lg}S_{i}\frac{\mathrm{lg}N_{0}-\mathrm{lg}N_{A}}{\mathrm{lg}N_{i}-\mathrm{lg}N_{A}}$$ (2-10)

where *N*_*A*_ is the low cycle fatigue life of the point A, *S*_*A*_ is the load amplitude determined through a linear least squares fit approach of the experiment data. Based on this approach, the distribution property of the fatigue strength can be determined to provide the basic parameters for the further comparative analysis.

## 3. Results

### 3.1 Prediction results based on the UKF

**Fig 4:**
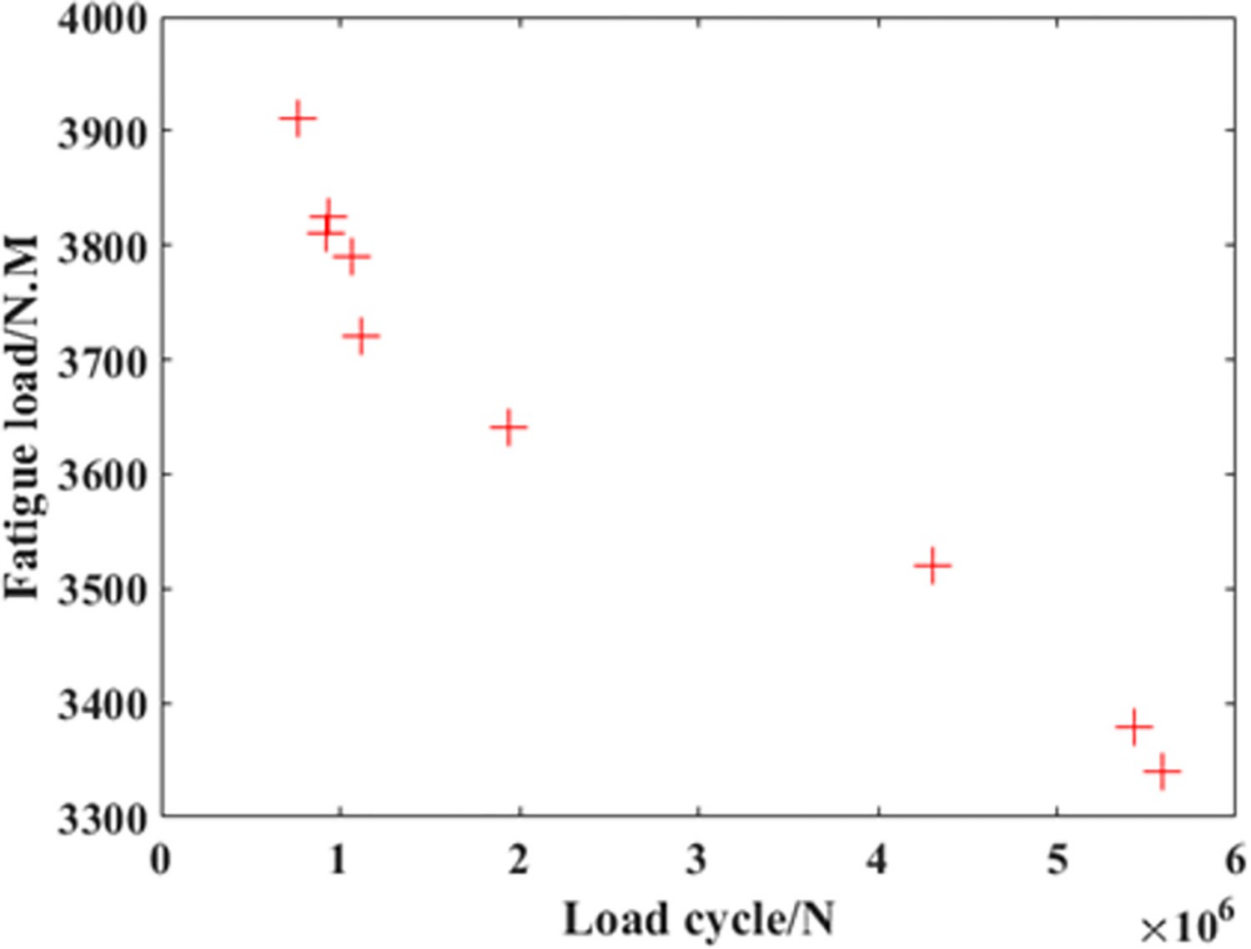
The experimental results of the crankshaft.

Based on the UKF mentioned in the previous chapter, it’s possible to predict the remaining fatigue life of the crankshaft during the experiment process. On the other hand, as introduced in the previous chapter, the experiment cost is nearly liner to the number of the load cycles applied on the crankshaft. For the crankshaft, the number of the load cycles in each case is among the range from 10^5^ to 10^7^. So in this paper, only the cases among which the load cycles was more than 10^6^ were selected to be the object of predicting. As shown in Fig 4, among the whole experiment results, three groups of data can fulfill this demand. So in this paper, these experiment results were chosen to be the object of prediction.

**Fig 5:**
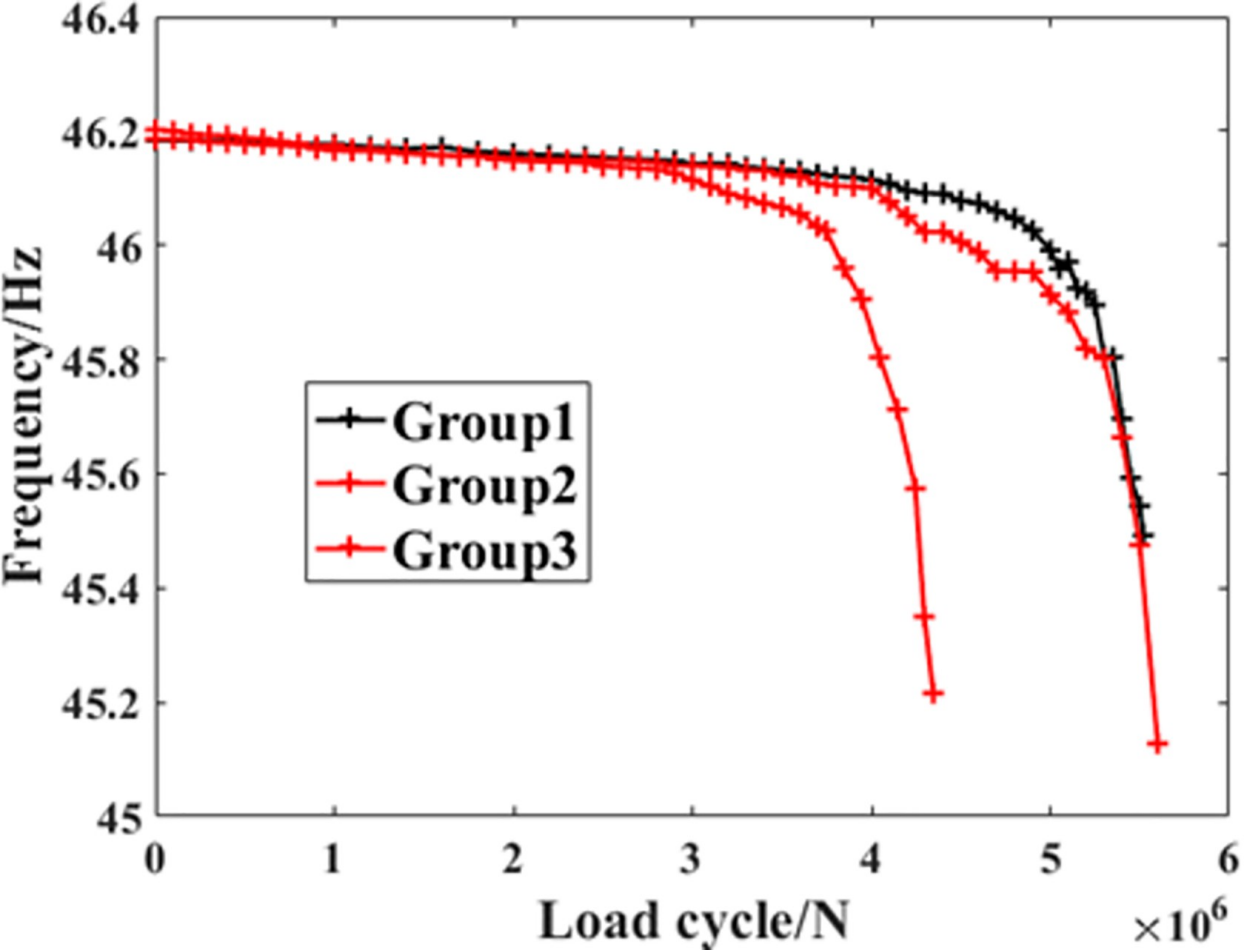
The changing process of the first order inherent frequency of the crankshafts.

As mentioned in the previous chapter, the failure criteria of the crankshaft is defined based on the reduction amount of first order inherent frequency of the system. Fig 5 shows the changing processes of this parameters in each cases, from which it can be discovered that the parameter drops rapidly when the reduction amount has reached 1 Hz, which is consistent in the experiment standard. Based on this fact, the residual fatigue life can be predicted by analyzing the changing process of the parameter before the final failure happens. In order to make our conclusion more comprehensive, three kinds of sampling ranges and the corresponding prediction ranges were selected to conduct the comparative study. The detailed definition of them are shown in Table 3.

**Table 3:** The definition of the different sampling and prediction ranges.

Sampling range			Prediction range		
Serial Range number	Start point	End point	Serial Range number	Start point	End point
Range 1	0Hz	0.2Hz	Range 1	0.2Hz	1Hz
Range 2	0Hz	0.3Hz	Range 2	0.3Hz	1Hz
Range 3	0Hz	0.4Hz	Range 3	0.4Hz	1Hz

**Fig 6:**
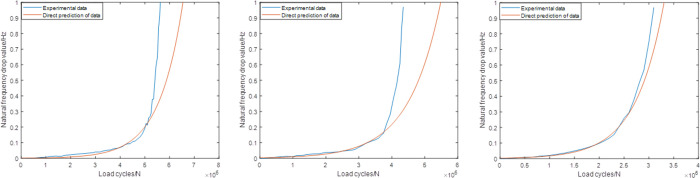
Predictions based on the Range1 (group1) (group2) (group3).

**Fig 7:**
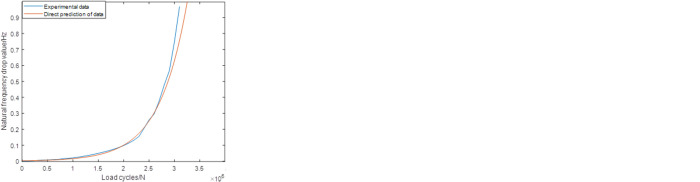
Predictions based on the Range2 (group1) (group2) (group3).

**Fig 8:**
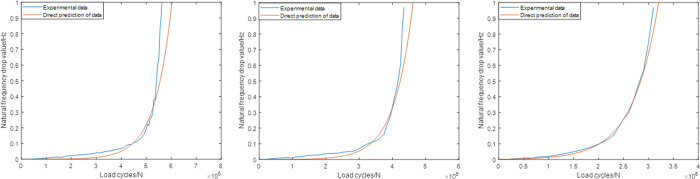
Predictions based on the Range3 (group1) (group2) (group3).

As shown in Table 3, the combination of the different sampling ranges and prediction ranges in each group are combined to make up the whole failure process. Figs 6–8 shows the prediction results of these groups. As shown in Fig 6, it’s not difficult to find that the predictions based on this sampling range may result in some relatively large errors, especially for group2 case (the relative error is about 30%). While for the predictions based on Range2, the accuracy has been improved obviously. For group1, group2 and group3, the values of the relative errors are less than 15%. For the predictions based on Range3, the highest accuracy are established, which makes the corresponding predictions more suitable for further statistical analysis.

Table 4 shows the percentage of each prediction ranges during the whole experiment process, from which it can be found that how much experiment time can be saved. As shown in this table, although the predictions based on Range3 can provide the highest accuracy, but the aim of timesaver is not obvious, only about 10% of the experiment time has been saved. So it’s necessary to propose the corresponding optimization method to predict the residual fatigue life as accurate as possible with relatively less time.

**Table 4:** The percentage of each prediction ranges during the whole experiment process.

Range 1		Range 2		Range 3	
Group number	Percentage	Group number	Percentage	Group number	Percentage
Group 1	27.1%	Group 1	11.1%	Group 1	9.2%
Group 2	28.6%	Group 2	13.8%	Group 2	8.9%
Group 3	35.5%	Group 3	25.8%	Group 3	16.1%
Average	30.4%	Average	16.9%	Average	11.4%

### 3.2 Model modification research

As shown in the above section, the precision of prediction was influenced by the size of the sampling range obviously, as well as the percentage of timesaver. The main reason for the errors generated during the predictions may be attributed to the crack growth speed. Since the beginning of the load stage, the fatigue crack initiates at the fillet of the crankpin and gradually grows until the final failure happens. This crack will also result in the reduction of the system stiffness, as well as the value of the failure parameter. On the other hand, the crack growth speed throughout the whole process is not steady. In this paper, the crankshaft material is 42CrMo. For the components made by this high strength alloy steel, the fatigue crack growth process is usually composed by three parts: the initiation period, the steady propagation period, and the quick growth period. In each stage, the propagation speed varies greatly from those among the other two stages [23,24]. This difference makes the prediction within different ranges may contains obvious errors.

In order to remove this hidden trouble in advance, the modified sampling ranges were proposed in this paper. Table 5 shows the detailed information of the modified sampling ranges, from which it can be found that the start point in each case was 0.1Hz, based on this pretreatment the crack propagation speed with this stage are generally steady, which is benefic for the further prediction application.

**Table 5:** The definition of the modified sampling and prediction ranges.

Sampling range			Prediction range		
Serial Range number	Start point	End point	Serial Range number	Start point	End point
Range 1	0.1Hz	0.2Hz	Range 1	0.2Hz	1Hz
Range 2	0.1Hz	0.3Hz	Range 2	0.3Hz	1Hz
Range 3	0.1Hz	0.4Hz	Range 3	0.4Hz	1Hz

**Fig 9:**
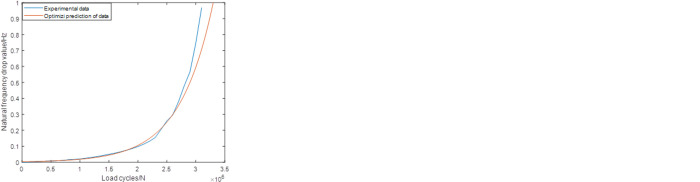
Predictions based on the modified Range1 (group1) (group2) (group3).

**Fig 10:**
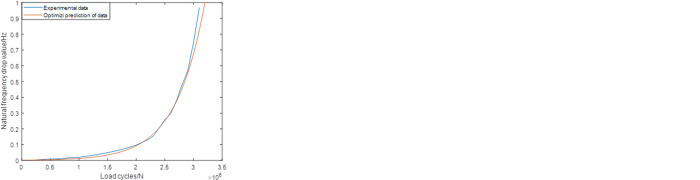
Predictions based on the modified Range2 (group1) (group2) (group3).

**Fig 11:**
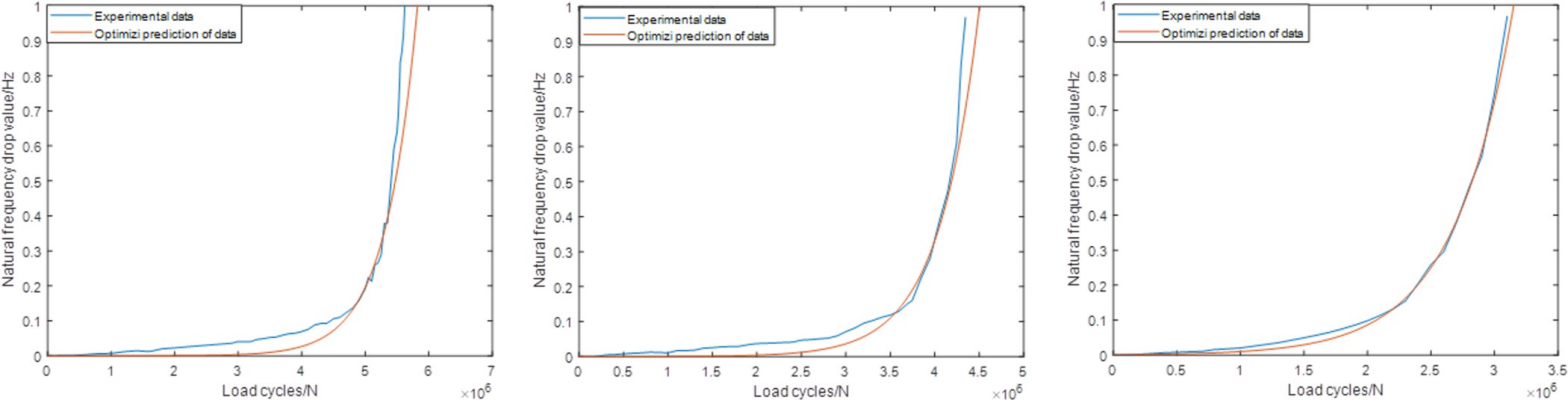
Predictions based on the modified Range2 (group1) (group2) (group3).

Figs 9–11 shows the predictions according to the newly proposed sampling ranges. As shown in Fig 9, compared with the original Range1, the modified Range1 provides more satisfactory results in all the prediction cases. The differences between the original experiment data and the predicted results has significantly decreased. For group1, group2 and group3, the errors are all less than 5%, which can already fulfill the engineering application demands. Fig 10 shows the predictions of all the three groups based on the modified Range2, which also shows obvious practicability in this condition. The errors in all the three groups are no more than 5%. For the same results based on the modified Range3, the highest accuracy are established, which makes the predictions and the actual experiment data nearly the same. Meanwhile, compared with the sampling ranges, the size of the prediction ranges based on different definitions remains unchanged, which makes the timesaver effect also unchanged in each cases.

In previous study, this modification method with the combination of the particle filtering algorithm was also applied in predicting the failure time node of the crankshaft during the loading stage. Table 6 shows the errors of the predictions based on these two approaches and the sampling Range1, from which it can be discovered that the particle filtering algorithm (PF) method may cause some relatively large errors in this application (the error may be more than 15%) within this sampling range, and the average error of the predictions based on UKF is much less than that based on PF, which makes this approach more suitable for the application.

**Table 6:** The errors based on different prediction approaches and Range1.

PF		UFKUKF	
Group number	Error	Group number	Error
Group1	4.3%	Group1	6.1%
Group2	4.1%	Group2	6.9%
Gropu3	16.6%	Gropu3	5.2%
Average	8%	Average	6.1%

### 3.3 Statistical analysis results

According to the research process introduced in the previous chapter, the key parameter in the fatigue property assessment of the crankshaft in actual application is the fatigue limit load under the specified survival rate (usually 50% or more). In this paper, the fatigue life prediction results were taken into the statistical analysis. Table 7 shows the media rank analysis results of the crankshaft based on different sampling ranges, from which it can be discovered that all the three groups of fatigue limit load distribution property based on different sampling ranges are very close to that based on the original test results. For the modified sampling Range1, over 30% of the experiment time can be saved to provide nearly the same analysis result for the fatigue limit load (the relative differences under all the media rank rates are lower than 1%). Table 8 shows the statistical analysis results based on the Gaussian distribution model, from which it can be found that the parameters are also nearly the same.

**Table 7:** The media rank estimation results based on different sources (*N·m*).

Original result	Range1	Range2	Range3	Media rank
4825	4834	4830	4829	0.1164
4879	4913	4898	4893	0.2195
4962	4992	4979	4974	0.2298
5024	5056	5044	5034	0.3421
5088	5114	5102	5098	0.4235
5128	5160	5143	5139	0.5568
5190	5211	5202	5199	0.6543
5201	5221	5213	5209	0.7689
5234	5262	5248	5249	0.8333
5415	5442	5430	5426	0.9379

**Table 8:** The main parameters of the fitting results based on the Gaussian distribution model (*N·m*).

Data source	*μ*	*σ*
Original result	5094	178
Range1	5120	179.2
Range2	5109	178.4
Range3	5105	178.9

## 4. Discussion and conclusion

High cycle bending fatigue property is the indispensable parameter in guiding the design and application of the crankshafts in modern engines. The paper proposed the accelerated test method for the application in the crankshaft high cycle bending fatigue test condition. The main conclusion of the research were draw as followed:

1) The precision of the residual fatigue life predictions based on the newly proposed sampling ranges are clearly much higher than that based on the conventional sampling ranges. The reason is that the advanced clearance effect of the experiment data within different damage stages provided by the modified sampling ranges.

2) The unscented Kalman filtering algorithm is superior to the particle filtering algorithm in predicting the time of fatigue failure during the crankshaft experiment process, which makes this approach more suitable for the application.

3) The smallest sampling range can save about 30% of the whole loading period time, meanwhile, affecting the key parameter (the fatigue limit load) slightly (lower than 1% or less), which makes the acceleration effect of this approach quite obvious, thus can be popularized and applied for the cost reduction of the experiment.

## Supporting information

S1 File Experiment data. (DOCX)
