# Supplementary material for: Research of the crankshaft high cycle bending fatigue experiment design method based on the modified unscented Kalman filtering algorithm and the SAFL approach
Source: PLoS One. 2023 Sep 12;18(9):e0291135. doi: 10.1371/journal.pone.0291135 (PMC10497137; doi:10.1371/journal.pone.0291135)
Supplement: S1 File — (DOCX) [file pone.0291135.s001.docx]

| Group1 | | Group 2 | | Group3 | |
| --- | --- | --- | --- | --- | --- |
| Load cycles | Frequency | Load cycles | Frequency | Load cycles | Frequency |
| 0 | 46.1837676 | 0 | 46.18536441 | 0 | 46.20421327 |
| 200000 | 46.18287768 | 100000 | 46.18535903 | 100000 | 46.2039167 |
| 400000 | 46.18260909 | 200000 | 46.18455358 | 200000 | 46.20163402 |
| 600000 | 46.17997484 | 300000 | 46.1804031 | 300000 | 46.19952764 |
| 800000 | 46.17723969 | 400000 | 46.17965963 | 400000 | 46.19707027 |
| 1000000 | 46.17673273 | 500000 | 46.17741531 | 500000 | 46.1959948 |
| 1200000 | 46.17199096 | 600000 | 46.17567437 | 600000 | 46.19446281 |
| 1400000 | 46.16902298 | 700000 | 46.17466319 | 700000 | 46.19315221 |
| 1600000 | 46.17135413 | 800000 | 46.17288514 | 800000 | 46.18811697 |
| 1800000 | 46.16345526 | 900000 | 46.17354025 | 900000 | 46.185992 |
| 2000000 | 46.160749 | 1000000 | 46.17453272 | 1000000 | 46.18344742 |
| 2200000 | 46.15765172 | 1100000 | 46.16838078 | 1100000 | 46.17868019 |
| 2400000 | 46.15477151 | 1200000 | 46.16813215 | 1200000 | 46.17367854 |
| 2600000 | 46.15180536 | 1300000 | 46.16681632 | 1300000 | 46.16838731 |
| 2800000 | 46.14887826 | 1400000 | 46.16140089 | 1400000 | 46.16150876 |
| 2900000 | 46.14743784 | 1500000 | 46.15982572 | 1500000 | 46.15453039 |
| 3000000 | 46.1426207 | 1600000 | 46.15725384 | 1600000 | 46.14691613 |
| 3100000 | 46.14317478 | 1700000 | 46.15656521 | 1700000 | 46.13878911 |
| 3200000 | 46.14253549 | 1800000 | 46.15583844 | 1800000 | 46.12910367 |
| 3300000 | 46.13638765 | 1900000 | 46.15104996 | 1900000 | 46.11832729 |
| 3400000 | 46.1343085 | 2000000 | 46.14809533 | 2000000 | 46.10581011 |
| 3500000 | 46.13111618 | 2100000 | 46.14714756 | 2100000 | 46.09063922 |
| 3600000 | 46.12967102 | 2200000 | 46.14610523 | 2200000 | 46.07273016 |
| 3700000 | 46.12421765 | 2300000 | 46.14494839 | 2300000 | 46.04901126 |
| 3800000 | 46.12016729 | 2400000 | 46.14393839 | 2400000 | 45.99812975 |
| 3900000 | 46.11853904 | 2500000 | 46.13817318 | 2500000 | 45.94524578 |
| 4000000 | 46.11414275 | 2600000 | 46.13670458 | 2600000 | 45.90857182 |
| 4100000 | 46.10785773 | 2700000 | 46.13444156 | 2700000 | 45.82560841 |
| 4200000 | 46.09569389 | 2800000 | 46.13241069 | 2800000 | 45.72536904 |
| 4300000 | 46.0910376 | 2900000 | 46.12619598 | 2900000 | 45.63643836 |
| 4400000 | 46.09010857 | 3000000 | 46.11394099 | 3000000 | 45.45662614 |
| 4500000 | 46.07774431 | 3100000 | 46.10365952 | 3100000 | 45.23606585 |
| 4600000 | 46.07355179 | 3200000 | 46.0899637 | 3200000 | 45.0322385 |
| 4700000 | 46.05988166 | 3300000 | 46.0823423 | 3300000 | 44.77197489 |
| 4800000 | 46.04713132 | 3400000 | 46.07336385 | 3400000 | 44.40971142 |
| 4900000 | 46.02611323 | 3500000 | 46.06660294 | 3500000 | 43.82957233 |
| 5000000 | 45.99123641 | 3600000 | 46.05530693 | 3550000 | 43.49804762 |
| 5050000 | 45.95911669 | 3700000 | 46.03256642 | 3600000 | 42.37456902 |
| 5100000 | 45.97108271 | 3746000 | 46.02536637 | 3650000 | 41.30750807 |
| 5150000 | 45.92402216 | 3846000 | 45.96059419 | 0 | 46.20421327 |
| 5200000 | 45.9182904 | 3946000 | 45.90563215 | 100000 | 46.2039167 |
| 5250000 | 45.89482514 | 4046000 | 45.80369294 | 200000 | 46.20163402 |
| 5300000 | 45.80546285 | 4146000 | 45.71277655 | 300000 | 46.19952764 |
| 5350000 | 45.80393362 | 4246000 | 45.57406384 | 400000 | 46.19707027 |
| 5400000 | 45.6962173 | 4296000 | 45.34987868 | 500000 | 46.1959948 |
| 5450000 | 45.59261612 | 4346000 | 45.21616643 | 600000 | 46.19446281 |
| 5500000 | 45.54347964 | 4396000 | 45.05684672 | 700000 | 46.19315221 |
| 5520000 | 45.49246754 | 4426000 | 44.96288595 | 800000 | 46.18811697 |
| 5540000 | 45.40408205 | 4446000 | 44.86251537 | 900000 | 46.185992 |
| 5550000 | 45.34584501 | 4476000 | 44.63677341 | 1000000 | 46.18344742 |
| 5575000 | 45.31680367 | 4496000 | 44.49319065 | 1100000 | 46.17868019 |
| 5600000 | 45.26973931 | 4521000 | 44.27051842 | 1200000 | 46.17367854 |
| 5625000 | 45.16713021 | 4546000 | 43.76659208 | 1300000 | 46.16838731 |
| 5650000 | 45.04880818 | 4571000 | 43.64575228 | 1400000 | 46.16150876 |
| 5675000 | 44.95109179 | 4596000 | 43.18523138 |  |  |
| 5700000 | 44.73784362 | 4621000 | 42.52749925 |  |  |
| 5725000 | 44.59015717 | 4642000 | 40.79620494 |  |  |
| 5750000 | 44.28440924 |  |  |  |  |
| 5775000 | 44.00977128 |  |  |  |  |
| 5800000 | 43.77605571 |  |  |  |  |
| 5825000 | 43.43893216 |  |  |  |  |
| 5835000 | 43.1182145 |  |  |  |  |
| 5845000 | 42.6939832 |  |  |  |  |
| 5850000 | 42.44419665 |  |  |  |  |
| 5855000 | 41.94889547 |  |  |  |  |
| 5860000 | 41.4803855 |  |  |  |  |
|  |  |  |  |  |  |
